# A multicenter study to assess the performance of the point-of-care RT-PCR Cobas SARS-CoV-2 & Influenza A/B nucleic acid test for use on the Cobas Liat system in comparison with centralized assays across healthcare facilities in the United States

**DOI:** 10.1128/jcm.01459-24

**Published:** 2025-07-02

**Authors:** Elissa M. Robbins, Rasa Bertuzis, Ho-Chen Chiu, Lupe Miller, Christopher Noutsios

**Affiliations:** 1 Roche Molecular Systems, Inc. 53527 , Pleasanton, California, USA; 2 Roche Sequencing Solutions, Inc., Indianapolis, Indiana, USA; Boston Children's Hospital, Boston, Massachusetts, USA

**Keywords:** point of care, performance, SARS-CoV-2, influenza A, influenza B, nasal, nasopharyngeal, primary care, outpatients, emergency department

## Abstract

Respiratory diseases can share many of the same symptoms, highlighting the need for timely and accurate differentiation to facilitate effective clinical management and reduce transmission. Compared with centralized testing, molecular point-of-care tests (POCTs) can provide a faster time to result. We evaluated the RT-PCR POCT Cobas® SARS-CoV-2 & Influenza A/B qualitative assay for use on the Cobas Liat system (the POC SARS-CoV-2 & Influenza A/B test) in nasal and nasopharyngeal swab samples from 10 diverse healthcare facilities in the United States. A composite comparator design consisting of three centralized tests was used to analyze severe acute respiratory syndrome coronavirus 2 (SARS-CoV-2), while performance vs a single centralized test was used for analysis of influenza A and B. Evaluations included performance stratified by sample type (prospective/retrospective and nasal/nasopharyngeal [paired by subject]), collection method (self/healthcare worker-collected [alternated and approximately balanced]), symptom status (symptomatic/asymptomatic), and SARS-CoV-2 vaccination status, as well as assay inclusivity and system ease of use. A total of 2,247 samples were tested. For SARS-CoV-2, the overall percent agreement (OPA) was 98.8% (95% confidence interval [CI]: 97.9, 99.3) in nasal swab samples and 99.0% (95% CI: 98.2, 99.4) in nasopharyngeal swab samples. Regression analysis showed that cycle threshold values from paired nasal and nasopharyngeal swab samples were highly correlated (correlation coefficient 0.83). The OPA was ≥99.5% (sample type dependent) and 100.0% for influenza A and B, respectively. The POC SARS-CoV-2 & Influenza A/B test was easy to use. These results support the use of the POCT in various sample types and by various operators in the intended-use setting.

**IMPORTANCE:**

This study highlights the benefits of RT-PCR point-of-care tests, namely comparable performance to centralized testing in multiple sample types and ease of use. Utilizing assays such as the POC Cobas SARS-CoV-2 & Influenza A/B test may improve the timely differentiation of respiratory diseases that share similar symptoms.

## INTRODUCTION

More than 4 years since the start of the coronavirus disease 2019 (COVID-19) pandemic, much of the world has returned to normality, yet COVID-19 continues to cause severe illness and mortality in the United States (US) and elsewhere (1, 2). This applies particularly to the immunocompromised, who are at risk of severe outcomes (3) and comprise approximately 3% of the US adult population (4). Disease activity is year-round, but seasonal spikes occur from November to April (5), coinciding with a circulation of other respiratory viruses, such as influenza (6). Respiratory diseases such as COVID-19 and influenza share similar symptoms (7), highlighting the need for timely and accurate differentiation of the causative agent to help facilitate effective clinical management and reduce onward transmission.

The current choice of testing for severe acute respiratory syndrome coronavirus 2 (SARS-CoV-2) is setting dependent (8), but standard laboratory-based (i.e., centralized) nucleic acid amplification tests are the mainstay of diagnosis (9). Numerous antigen or molecular point-of-care tests (POCTs) are commercially available (10, 11), offering the benefit of a faster time to result (12). Both POCT formats have lower performance compared with centralized testing, particularly sensitivity (13)—a secondary analysis of antigen POCT found the performance to be commonly lower than stated within manufacturers’ instructions for use (14)—but molecular POCTs have relatively higher performance in comparison with antigen POCTs, with pooled sensitivity values of 93% (99% specificity) vs 71% (99% specificity), respectively (13). Molecular POCTs can provide actionable results in as little as 20 minutes depending on the assay and workflow (11).

Influenza is a significant cause of severe illness and death (15–17), resulting in a substantial clinical burden to patients and the healthcare system alike (18). Molecular POCTs for the detection of influenza A/B subtypes and other respiratory viruses such as respiratory syncytial virus have been available for several years (11, 12, 19–22). Clinical practice guidelines from the Infectious Diseases Society of America recommend that antivirals are initiated for certain patient groups as soon as possible regardless of illness duration, or within 48 hours of illness onset (23), supporting the use of rapid molecular POCTs for influenza testing in preference to centralized laboratory testing. In the hospital setting, an interventional study of a molecular POCT for influenza found significant improvements in isolation practices and reductions in length of stay compared with centralized testing (24), while a modeling study found that the introduction of POCTs could reduce time to diagnosis, hospital stay, and in-hospital costs (25). Molecular POCTs for influenza can generate sensitivity estimates of ≥96%–100% in real-world settings (22, 26).

The primary objective of this study was to evaluate the real-world clinical performance of the POC Cobas® SARS-CoV-2 & Influenza A/B qualitative assay for use on the Cobas Liat system (here referred to as the POC SARS-CoV-2 & Influenza A/B), a multiplexed RT-PCR test combining measurement of SARS-CoV-2 and influenza subtypes, in reference to comparator centralized PCR tests for SARS-CoV-2 and influenza A and B viruses. The sample types were nasal and nasopharyngeal swab specimens from individuals with suspected respiratory viral infection consistent with COVID-19 and asymptomatic individuals exposed to COVID-19, who presented at healthcare facilities across the US during the COVID-19 public health emergency. The secondary objectives were to evaluate the influence of the collection method and the ease of use of the Cobas Liat system.

## MATERIALS AND METHODS

### Sites

Testing was conducted at 11 sites: 10 geographically diverse healthcare facilities that were representative of intended-use sites (i.e., POC settings, such as emergency departments, urgent care, pediatric and primary care clinics, and drive-through COVID-19 testing sites), and one reference laboratory for centralized testing. After testing using the POC SARS-CoV-2 & Influenza A/B test at the healthcare facilities, samples were shipped to the reference laboratory for analysis with the comparator assays (see below).

Healthcare facilities were selected based on sample availability, ability to provide adequate resources and operators, ability to adhere to good clinical practice, and with the aim of ensuring representation of the demographic diversity of the US population. Clinical Laboratory Improvement Amendments (CLIA) certification was not a requirement for the study; therefore, not all healthcare facilities performing testing were certified under CLIA regulations to perform waived testing, but all facilities met the requirements for CLIA-waived settings (i.e., intended-use settings using untrained non-laboratory personnel). All healthcare facilities performed both sample collection and POC testing. POC operators at the 10 healthcare facilities (*n* = 30 operators in total) had limited or no laboratory training and were representative of typical test operators in CLIA-waived settings (e.g., nurses, nursing assistants, and medical assistants). Operators at the reference laboratory were blinded to the results generated at the healthcare facilities.

### Study population

The symptomatic group comprised samples from individuals with suspected respiratory viral infection consistent with COVID-19 (see Signs/symptoms, Supplemental material). The asymptomatic group comprised individuals with self-reported “recent” exposure (timeframe undefined) to SARS-CoV-2-positive individuals or any other reasons to suspect COVID-19. Patient clinical information was gathered, including, but not limited to, antiviral usage for up to 7 days prior to and on the day of sample collection, gender, race/ethnic group, and age. Individuals presenting at the healthcare facilities were considered for study inclusion based on eligibility criteria (see Eligibility criteria, Supplemental material).

### Study design

The clinical performance of the POC SARS-CoV-2 & Influenza A/B test (27) was evaluated by comparing results to a composite comparator method for the SARS-CoV-2 analyte using the following three highly sensitive centralized testing assays: Cobas SARS-CoV-2 qualitative assay for use on the Cobas 6800/8800 systems (here referred to as the 6800/8800 SARS-CoV-2 test) (28); Cobas SARS-CoV-2 & Influenza A/B qualitative assay for use on the Cobas 6800/8800 systems (here referred to as the 6800/8800 SARS-CoV-2 & Influenza A/B test) (29); and Hologic Aptima SARS-CoV-2 Assay (30).

The clinical performance for the influenza A/B analyte components of the POC SARS-CoV-2 & Influenza A/B test was evaluated against a single centralized testing assay, the 6800/8800 SARS-CoV-2 & Influenza A/B (29).

Samples that generated invalid results were repeated where sample volume permitted additional testing; samples generating a second invalid result upon retesting were reported as invalid. Interrogation of discrepant samples was not performed, though selected samples were further retested with the POC SARS-CoV-2 & Influenza A/B test for exploratory purposes.

### Sample types and collection

Sample types included fresh and frozen nasal and nasopharyngeal swab samples, collected in the same media formulations but under different brand names: Copan Universal Transport Medium (UTM) or BD Universal Viral Transport (UVT) medium.

#### Prospective fresh samples (UTM)

One nasal swab and one nasopharyngeal swab sample were collected from each study participant. A nasal swab of both nostrils was first collected either by the healthcare worker (HCW), here referred to as “HCW-collected,” or by the study participant under instruction from the HCW, here referred to as “self-collected,” as per manufacturer’s instructions (31). A nasopharyngeal swab was then collected by HCWs from the same study participant to generate paired samples. If a nasopharyngeal swab had already been collected (using one nostril) as part of the healthcare facility’s standard of care, the study nasopharyngeal swab was taken from the opposite nostril.

All prospective samples were collected from patients presenting to healthcare facilities during February to June 2022, characteristic of the 2021/2022 winter respiratory season.

#### Retrospective frozen samples (archived or purchased from external vendors; UTM or UVT)

The low influenza prevalence in the prospective population was anticipated because of reduced international travel, new respiratory care paradigms, increased public health awareness, and use of interventions (such as masking policies or social distancing) that occurred during the COVID-19 pandemic and study enrollment period, in which influenza A virus had been the dominant virus type in circulation (32). Therefore, frozen samples positive for influenza A virus and for influenza B virus were used to supplement the prospectively collected fresh samples. These retrospective samples were collected in the US during the 2013–2014, 2014–2015, and 2019–2020 influenza seasons. Basƒeline demographic data and patient characteristics related to these samples were unavailable.

Retrospective samples with known SARS-CoV-2 status, collected between 29 March 2021 and 26 May 2021, were also included in the SARS-CoV-2 analyses.

Whilst multiple freeze-thaw cycles have a limited effect on the viral titer (33), there is the potential for samples that were initially low-titer positives to appear negative following storage. Internal Roche data support the stability of viral titer for up to three freeze-thaw cycles, so retrospective samples were only eligible for inclusion if they had undergone no more than two freeze-thaw cycles. Retrospective positive and negative samples were blended in a standard POCT workflow and assessed alongside prospectively collected samples.

### Details of assays and instruments

#### POC SARS-CoV-2 & Influenza A/B test (POC RT-PCR)

The Cobas Liat analyzer is for *in vitro* diagnostic use (27). The analyzer automates all nucleic acid amplification test processes, including target enrichment, inhibitor removal, nucleic acid extraction, reverse transcription, DNA amplification, real-time detection, and result interpretation in approximately 20 minutes (27). The Cobas Liat system comprises the Cobas Liat analyzer in conjunction with the Cobas Liat assay tubes (27). At the limit of detection for this system, representing the lowest concentration at which ≥95% of samples are accurately detected, the mean Liat cycle threshold (Ct) value was 33.2.

The POC SARS-CoV-2 & Influenza A/B test utilizes a single-use disposable assay tube that contains all the reagents necessary for the detection of SARS-CoV-2 and hosts the sample preparation and PCR processes (34). This multiplex real-time RT-PCR test is intended for the simultaneous rapid *in vitro* qualitative detection and differentiation of SARS-CoV-2, influenza A, and influenza B virus RNA in healthcare provider-collected nasal and nasopharyngeal swab samples and self-collected nasal swab samples (collected in a healthcare setting with instruction by a healthcare provider) from individuals suspected of respiratory viral infection consistent with COVID-19 (34).

#### 6800/8800 SARS-CoV-2 & Influenza A/B test (centralized testing method 1)

The Cobas 6800/8800 systems consist of the sample supply module, the transfer module, the processing module, and the analytic module (29). Automated data management is performed by the Cobas 6800/8800 software, which assigns results for all tests. Results are available in less than 3.5 hours after loading the sample on the system (29). The positive SARS-CoV-2 result is defined as positive on the SARS-CoV-2 channel (target 2) and/or the pan-sarbeco channel (target 3).

The 6800/8800 SARS-CoV-2 & Influenza A/B test is an automated multiplex real-time RT-PCR assay intended for simultaneous qualitative detection and differentiation of SARS-CoV-2, influenza A virus, and/or influenza B virus RNA in healthcare provider-collected nasal and nasopharyngeal swab samples and self-collected nasal swab samples (collected in a healthcare setting with instruction by a healthcare provider) from individuals suspected of respiratory viral infection consistent with COVID-19 (29).

#### 6800/8800 SARS-CoV-2 test (centralized testing method 2)

The 6800/8800 SARS-CoV-2 test is an automated real-time RT-PCR assay intended for the qualitative detection of nucleic acids from SARS-CoV-2 in healthcare provider-instructed, self-collected anterior nasal (nasal) swab samples (collected onsite) and healthcare provider-collected nasal, nasopharyngeal, and oropharyngeal swab samples collected from any individuals, including those suspected of COVID-19 by their healthcare provider and those without symptoms or with other reasons to suspect COVID-19 (28). The positive SARS-CoV-2 result is defined as positive on the SARS-CoV-2 channel (target 1) and/or the pan-sarbeco channel (target 2).

#### Hologic Aptima SARS-CoV-2 assay (tiebreaker; centralized testing method 3)

As a commonly used molecular assay (30), centralized testing method (CTM) 3 was utilized as a tiebreaker. The test utilizes transcription-mediated amplification for the qualitative detection of RNA from SARS-CoV-2 isolated and purified from nasopharyngeal, nasal, mid-turbinate and oropharyngeal swab samples, nasopharyngeal wash/aspirate, or nasal aspirates obtained from individuals meeting COVID-19 clinical and/or epidemiological criteria (35).

### Analyses

All data analyses were performed using SAS/STAT software (v9.4 or higher of the SAS System for Linux). No formal sample size calculations were calculated for this study, but patient enrollment was adjusted to accommodate disease prevalence and to generate a minimum of 50 SARS-CoV-2 positives, 30 influenza A positives, and 10 influenza B positives for analysis.

The clinical performance of the POC SARS-CoV-2 & Influenza A/B test was evaluated using estimates of positive percent agreement (PPA), negative percent agreement (NPA), and overall percent agreement (OPA), calculated with two-sided 95% confidence intervals (CIs) using the Wilson-score method (36). Comparison was against either the composite comparator for SARS-CoV-2 (CTM 1, CTM 2, and CTM 3 [tiebreaker if required]) or a single comparator for influenza A/B (CTM 1).

For SARS-CoV-2, concordant results from CTM 1 and CTM 2 established true positive or true negative status. In the event of discordance between CTM 1 and CTM 2, CTM 3 (the tiebreaker) was used to establish true status. Samples could be coded as uninterpretable in the event of invalid, failed, aborted, or missing results that were not resolved upon retesting. If CTM 1, CTM 2 or CTM 3 returned uninterpretable results, and the other two methods returned different results (i.e., CTM 1 positive, CTM 2 negative, and CTM 3 uninterpretable), then the status of the sample would be described as indeterminate.

McNemar’s mid-*P* test (*P*-value) was used to assess any differences between sample types. A *P*-value of <0.05 was considered statistically significant.

POCT operators were asked to complete a questionnaire to evaluate the ease of use of the POC SARS-CoV-2 & Influenza A/B test.

*In silico* analysis of the POC SARS-CoV-2 & Influenza A/B test was performed to assess assay design inclusivity on all available SARS-CoV-2 sequences (taxonomy ID 2697049) in the Global Initiative on Sharing All Influenza Data (GISAID) and National Center for Biotechnology Information (NCBI) databases up to July 2024 (>16,800,000 sequences in NCBI and >8,900,000 sequences in GISAID). The predicted impact of each variant on gene target 1 and gene target 2 primer and probe binding site sequence was evaluated using Roche proprietary software and Melting5 software. A SARS-CoV-2 sequence would potentially not be detected by the POC SARS-CoV-2 & Influenza A/B test if a delay in cycle threshold greater than five cycles and/or a probe melting temperature of <65°C was predicted.

## RESULTS

### Participants and summary of testing results

Subjects from 10 geographically diverse healthcare facilities were enrolled in the study. The summary of testing results can be seen in Table S1. A total of 2,209 SARS-CoV-2 results from prospective samples (both nasal and nasopharyngeal swabs) were valid for inclusion in downstream analyses.

### Population and baseline characteristics

Baseline demographic data for study participants were available for the prospectively collected samples; details on the study participants are presented in Table 1. Of these subjects, 506 were male (47.8%) and 553 were female (52.2%). The median age was 35 years (range 0–86).

**TABLE 1:** Demographics (prospective symptomatic and asymptomatic subjects)

Characteristics	Prospective population
Total	
*N*	1,059
Age (years)	
Mean	34.2
SD	19.88
Median	35.0
Range (minimum−maximum)^*a*^	0.0–86.0
Age group (years), *n* (%)	
≤18	287 (27.1)
19–39	319 (30.1)
40–64	391 (36.9)
≥65	62 (5.9)
Sex at birth, *n* (%)	
Male	506 (47.8)
Female	553 (52.2)
Ethnicity, *n* (%)	
Hispanic/Latino	212 (20.0)
Not Hispanic/not Latino	589 (55.6)
Not reported^*b*^	236 (22.3)
Unknown^*c*^	22 (2.1)
Race, *n* (%)	
American Indian/Alaskan Native	3 (0.3)
Asian	37 (3.5)
Black/African American	175 (16.5)
Native Hawaiian/Pacific Islander	6 (0.6)
White	520 (49.1)
Other	56 (5.3)
Not reported^*b*^	262 (24.7)

^*a*^ All subjects <1 year old are counted as age 0.

^*b*^ A clinical site that was a mobile drive-through site was not able to collect race/ethnicity identification from subjects.

^*c*^ Unknown category indicates subjects for whom the corresponding information is not available.

Patient characteristics are present in Table S2. In total, 60.4% of subjects in the prospective population had signs and symptoms of respiratory infection, with days from onset of first symptom ranging from 1 to 365 days. The remaining 39.6% of subjects were asymptomatic but were clinically suspected of SARS-CoV-2 infection by their healthcare provider due to recent exposure or other reason. The nasal swab samples were evenly distributed between those collected by HCW and those that were self-collected.

### Medical history

The medical history relating to vaccination status is presented in Table 2. Most participants received a COVID-19 vaccine (68.7%), with Pfizer as the most common vaccine for both first and second doses (69.5% and 72.2%, respectively).

**TABLE 2:** Subject medical history (prospective symptomatic and asymptomatic subjects)

Characteristics	Population statistics
Total	
*N*	1,059
Subject received the influenza vaccine within the last 6 weeks, *n* (%)	
Yes	14 (1.3)
No	1,045 (98.7)
Unknown	0 (0.0)
Influenza vaccine route of administration, *n* (%)^*a*^	
Intramuscular	14 (100.0)
Intranasal	0 (0.0)
Unknown	0 (0.0)
Subject received a COVID-19 vaccine, *n* (%)	
Yes	728 (68.7)
No	303 (28.6)
Unknown	28 (2.6)
Type of COVID-19 vaccine received for first dose, *n* (%)^*b*^	
Pfizer	506 (69.5)
Moderna	185 (25.4)
Johnson and Johnson (J & J)	30 (4.1)
Other	3 (0.4)
Unknown	4 (0.5)
Type of COVID-19 vaccine received for second dose, *n* (%)^*b*^	
Pfizer	488 (72.2)
Moderna	182 (26.9)
Other	3 (0.4)
Unknown	3 (0.4)
Type of COVID-19 vaccine received first booster, *n* (%)^*b*^^,*c*^	
Pfizer	217 (64.0)
Moderna	115 (33.9)
Johnson and Johnson (J & J)	5 (1.5)
Other	1 (0.3)
Unknown	1 (0.3)

^*a*^ Percentages calculated based on number of subjects who received the influenza vaccine within the last 6 weeks prior to enrollment and sample collection.

^*b*^ Percentages calculated based on number of subjects who received COVID-19 vaccine or first booster.

^*c*^ There were six subjects that reported receiving a second booster.

### SARS-CoV-2

#### Determination of the composite comparator status for SARS-CoV-2

In symptomatic study participants, the composite comparator method was SARS-CoV-2 positive for 134 nasal and 131 nasopharyngeal swab samples (Table S3).

In asymptomatic study participants, the composite comparator method was SARS-CoV-2 positive for 39 nasal and 39 nasopharyngeal swab samples (Table S3).

#### Performance

**Fig 1:**
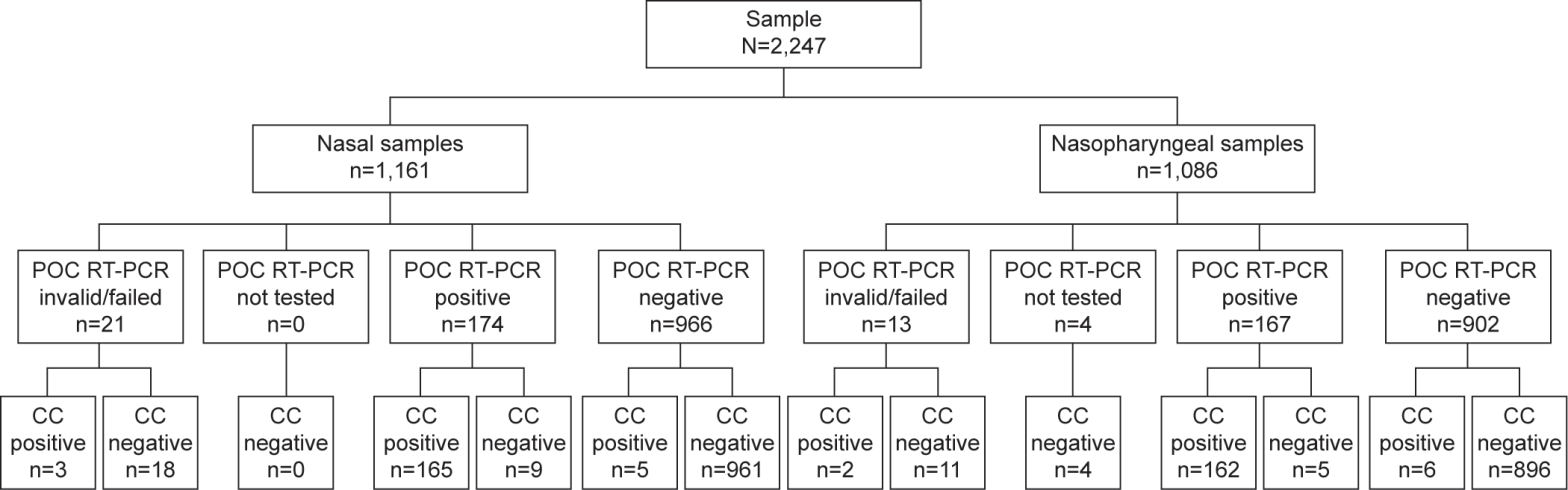
Detection of SARS-CoV-2 using the POC SARS-CoV-2 & Influenza A/B test and the composite comparator (prospective and retrospective, symptomatic and asymptomatic subjects). The composite comparator comprises the 6800/8800 SARS-CoV-2 & Influenza A/B test, 6800/8800 SARS-CoV-2 test, and Hologic Aptima SARS-CoV-2 Assay; the POC RT-PCR test is the POC SARS-CoV-2 & Influenza A/B test. The Cobas SARS-CoV-2 & Influenza A/B nucleic acid test for use on the Cobas Liat system is here referred to as the POC SARS-CoV-2 & Influenza A/B test. The Cobas SARS-CoV-2 & Influenza A/B qualitative assay for use on the Cobas 6800/8800 systems is here referred to as the 6800/8800 SARS-CoV-2 & Influenza A/B test. Cobas SARS-CoV-2 qualitative assay for use on the Cobas 6800/8800 systems is here referred to as the 6800/8800 SARS-CoV-2 test. Abbreviations: CC, composite comparator; POC RT-PCR, point-of-care RT-PCR.

The total number of samples that were positive, negative, or invalid, and how these align with the composite comparator status, can be seen in Fig. 1. The diagnostic performance (total number of positive and negative results) for SARS-CoV-2 can be seen in Table 3. Agreement between the composite comparator and the POC SARS-CoV-2 & Influenza A/B test for detection of SARS-CoV-2 in nasal swabs (total, prospective and retrospective, self-collected or HCW-collected), nasopharyngeal swabs (total, prospective and retrospective), in samples from vaccinated and unvaccinated participants, and in symptomatic and asymptomatic participants can be seen in Table S4. For nasal swab samples, the total OPA, PPA, and NPA were 98.8% (95% CI: 97.9, 99.3), 97.1% (95% CI: 93.3, 98.7), and 99.1% (95% CI: 98.2, 99.5), respectively. Of note, the OPA for HCW-collected nasal swab samples was 98.4% (95% CI: 97.0, 99.2) and 99.1% (95% CI: 98.0, 99.6) for self-collected nasal swab samples. The total OPA, PPA, and NPA for nasopharyngeal swab samples were 99.0% (95% CI: 98.2, 99.4), 96.4% (95% CI: 92.4, 98.4), and 99.4% (95% CI: 98.7, 99.8), respectively.

**TABLE 3:** Results for the detection of SARS-CoV-2 using the composite comparator compared with the POC SARS-CoV-2 & Influenza A/B test^*a*^

	Composite comparator (+)	Composite comparator (−)	Total	PPA % (95% CI)	NPA % (95% CI)
Nasal					
POC SARS-CoV-2 & Influenza A/B test (+)	165	9	174	**97.1 (93.3, 98.7)**	**99.1 (98.2, 99.5)**
POC SARS-CoV-2 & Influenza A/B test (−)	5	961	966		
Total	**170**	**970**	**1,140**		
Nasopharyngeal					
POC SARS-CoV-2 & Influenza A/B test (+)	162	5	167	**96.4 (92.4, 98.4)**	**99.4 (98.7, 99.8)**
POC SARS-CoV-2 & Influenza A/B test (−)	6	896	902		
Total	**168**	**901**	**1,069**		

^*a*^ The Cobas SARS-CoV-2 & Influenza A/B nucleic acid test for use on the Cobas Liat system is here referred to as the POC SARS-CoV-2 & Influenza A/B test.

Using all paired results for nasal and nasopharyngeal swab samples by subject, the swab type did not make a difference in the reported result (McNemar’s mid-*P* test, *P* = 0.771).

#### Ct value distributions

**Fig 2:**
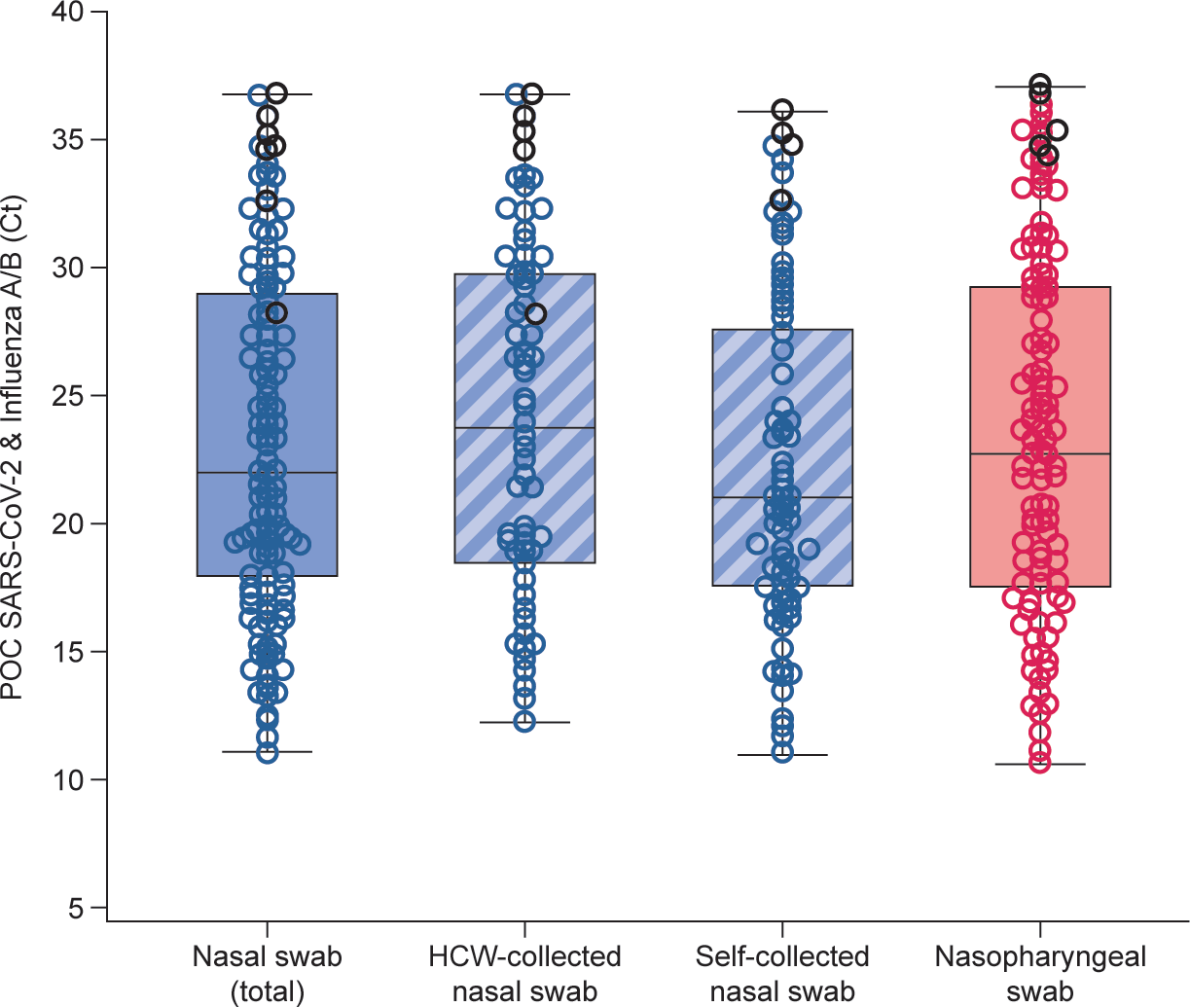
Ct values for the SARS-CoV-2 analyte from the POC SARS-CoV-2 & Influenza A/B test in nasal and nasopharyngeal swab samples. False positives are represented by black circles. Cross represents mean Ct values. Nasal (total): *n* = 179, mean Ct 23.1, median Ct 21.9, minimum Ct 11.0, and maximum Ct 36.8; HCW-collected nasal: *n* = 87, mean Ct 23.9, median Ct 23.7, minimum Ct 12.3, and maximum Ct 36.8; self-collected nasal: *n* = 92, mean Ct 22.4, median Ct 21.1, minimum Ct 11.0, and maximum Ct 36.1; nasopharyngeal: *n* = 177, mean Ct 23.4, median Ct 22.8, minimum Ct 10.6, and maximum Ct 37.1. False positives are indicated by black circles. The Cobas SARS-CoV-2 & Influenza A/B nucleic acid test for use on the Cobas Liat system is here referred to as the POC SARS-CoV-2 & Influenza A/B test. The horizontal solid lines represent median values. The upper and lower boundaries of the box plot represent the 75th and 25th percentiles.

The range of Ct values in nasal and nasopharyngeal swab samples can be seen in Fig. 2. Self-collected nasal swabs showed the highest average viral load, followed by nasopharyngeal swabs, then HCW-collected nasal swabs.

**Fig 3:**
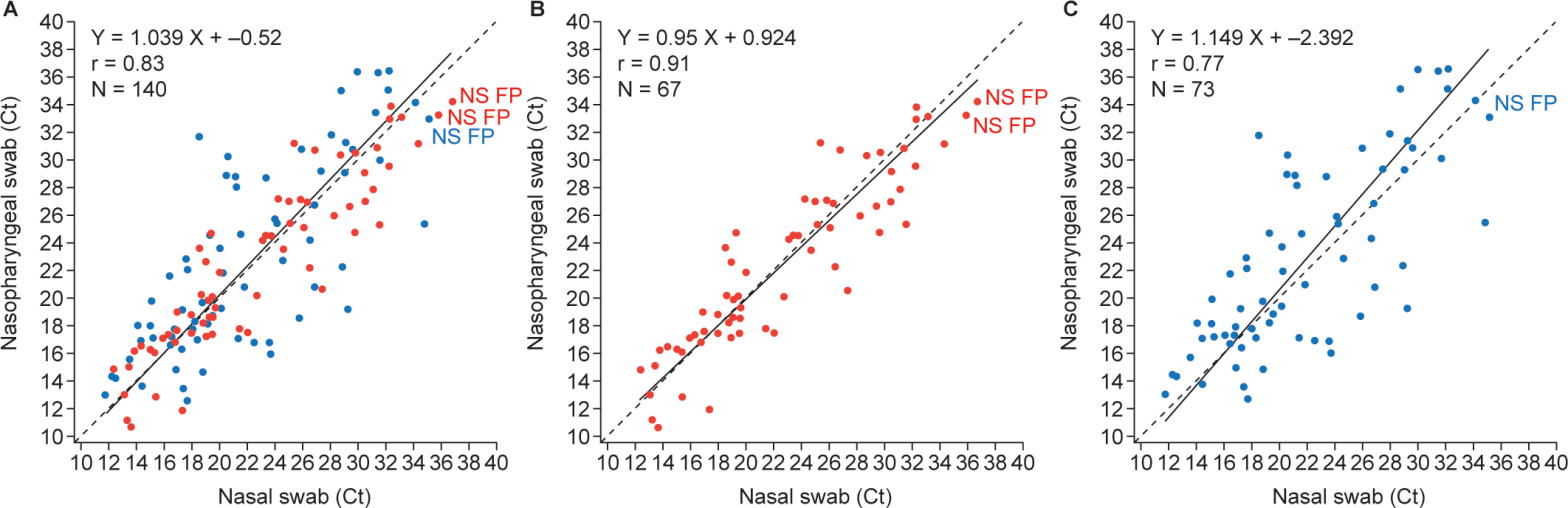
Deming regression for the SARS-CoV-2 analyte from the POC SARS-CoV-2 & Influenza A/B test in subject-paired nasal and nasopharyngeal swab samples. (A) Nasal (total). (B) HCW-collected nasal swab. (C) Self-collected nasal swab. Abbreviations: FP, false positive; NPS, nasopharyngeal swab; NS, nasal; NS FP, nasal false positive. The Cobas SARS-CoV-2 & Influenza A/B nucleic acid test for use on the Cobas Liat system is here referred to as the POC SARS-CoV-2 & Influenza A/B test. Prospective symptomatic = open circle; prospective exposed = triangle. The dashed line represents perfect correlation. The solid line represents Deming regression. Red data points represent HCW-collected nasal swabs. Blue data points represent self-collected nasal swabs.

The range of Ct values in subject-paired nasal and nasopharyngeal swab samples can be seen in Fig. 3. The Deming regression analysis showed that the two sample types were highly correlated (Fig. 3A; *r* = 0.83). Exploration of the collection method showed that HCW-collected nasal swabs had greater concordance between the paired samples (Fig. 3B; *r* = 0.91) than the self-collected nasal swabs (*r* = 0.77; Fig. 3C).

#### Discordance

Fourteen nasal and 11 nasopharyngeal swab samples showed discordant results between the POC SARS-CoV-2 & Influenza A/B test and the centralized testing methods (Table S5); 10 were positive only on the POC SARS-CoV-2 & Influenza A/B test.

### Influenza A

#### Performance

The agreement between the POC SARS-CoV-2 & Influenza A/B test and the 6800/8800 SARS-CoV-2 & Influenza A/B test for detection of influenza A in nasopharyngeal swabs (total, prospective and retrospective) and nasal swabs (total, prospective and retrospective, HCW-collected or self-collected) can be seen in Table S6. The OPA ranged from 98.9% to 100.0%.

#### Discordance

Two nasal and five nasopharyngeal swab samples showed discordant results between the POC SARS-CoV-2 & Influenza A/B test and the 6800/8800 SARS-CoV-2 & Influenza A/B test for detection of influenza A, four of which were positive only on the POC SARS-CoV-2 & Influenza A/B test. Ct values for 5/7 discordant samples ranged from 32.8 to 39.9 (near or above the limit of detection), while the remaining two had Ct values of 15.5 and 19.2 (Table S7).

### Influenza B

#### Performance

The agreement between the POC SARS-CoV-2 & Influenza A/B test and the 6800/8800 SARS-CoV-2 & Influenza A/B test for detection of influenza B in nasopharyngeal swabs (total, prospective and retrospective) and nasal swabs (total, prospective and retrospective, self-collected nasal or HCW-collected) can be seen in Table S8. The OPA for all comparisons was 100.0%.

#### Discordance

No discordant results between the POC SARS-CoV-2 & Influenza A/B test and the 6800/8800 SARS-CoV-2 & Influenza A/B test for detection of influenza B were recorded.

### Evaluation of the ease of use for the Cobas Liat system

Twenty-seven operators with experience in testing samples on the Cobas Liat system completed the questionnaire. The operators’ average scores indicating their agreement with the statements in the questionnaire are shown in Table S9. The overall score was 4.5 out of 5 for the operators’ answers to all eight statements, indicating that the operators agreed that the Cobas Liat system was easy to use.

### Inclusivity analysis

The POC SARS-CoV-2 & Influenza A/B test targets the nucleocapsid (N) and ORF1a/b regions of the SARS-CoV-2 genome (34). *In silico* analysis showed that 99.98% of NCBI and 99.99% of GISAID sequences for SARS-CoV-2 had no changes in the primer/probe binding sites of both target regions simultaneously. All sequences were predicted to be detected by at least one of the two sites.

## DISCUSSION

In this study, we evaluated the real-world clinical performance of the POC SARS-CoV-2 & Influenza A/B test against up to three centralized assays, stratified by various parameters. The high performance of the POC SARS-CoV-2 & Influenza A/B test in comparison with centralized testing methods utilizing nasopharyngeal or nasal samples has previously been reported (37–41), but this study is the first to report the performance of the POC SARS-CoV-2 & Influenza A/B test across variables such as collection method (self/HCW, retrospective/prospective), vaccination status, symptom status, and ease of use. Using the comparator result to establish the status of infection, we found high agreement between the POC RT-PCR test and the centralized testing assays for all three analytes across all variables.

Previously, a meta-analysis of nucleic acid amplification testing reported that the sensitivity for the detection of SARS-CoV-2 in nasal samples may be less than nasopharyngeal samples (42), while a study specifically assessing the POC SARS-CoV-2 & Influenza A/B test reported similar differences in SARS-CoV-2 detection by sample type (37). However, we saw no evidence of a sample-type difference in sensitivity, with SARS-CoV-2 positivity identified in both nasal and nasopharyngeal swab samples to a similar extent (POC SARS-CoV-2 & Influenza A/B test positivity rate approximately 15%; the difference in sensitivity [nasal minus nasopharyngeal] was +0.9%).

A small study from Denmark found almost equivalent sensitivity for detection of SARS-CoV-2 using self- and HCW-collected samples (84.2% vs 89.5%, respectively), and that patients preferred self-collection (43). In our study, whether nasal samples were collected by HCWs or by the patients themselves had little impact on the agreement values between the POC RT-PCR test and the comparators. Indeed, in all comparisons, the agreement for self-collected samples was actually higher than for HCW-collected samples. For SARS-CoV-2, Ct values of paired nasal and nasopharyngeal swab samples as measured by the POC RT-PCR test were highly correlated, and Ct values were on average lower in nasal swab samples (i.e., higher viral RNA titer) compared with nasopharyngeal swab samples, with self-collected nasal swabs exhibiting the lowest Ct values. Collectively, this indicates that the high performance level of the POC RT-PCR test is maintained across collection methods, and patients are able to self-sample effectively and are willing to do so.

We identified a small number of discrepant samples for the SARS-CoV-2 analyte in both nasal and nasopharyngeal swab samples, consistent with other studies (37, 38), most of which were false positives. The majority of the false positives were in the high Ct/low viral titer range (POC SARS-CoV-2 & Influenza A/B test Ct values: lowest 28.2, median 35.2, and highest 37.1) and thus close to or at the lower limit of detection of Ct 35.2 (USA-WA1/2020 strain) for the POC SARS-CoV-2 & Influenza A/B test (34). Of the false negatives detected by the POC SARS-CoV-2 & Influenza A/B test, all except one sample were in the high Ct range. Indeed, after exploratory re-testing of false-negative nasopharyngeal samples, two were found to be positive (re-tested Ct values were 34.8 and 35.4). Generally, low viral burden can be a cause of discrepant results (44), and a small external quality assessment (reproducibility) study reported that the performance of the POC SARS-CoV-2 & Influenza A/B test is highest at low Ct values (45).

US legislation categorizes tests for complexity (moderate or high) using seven criteria, such as the need for training and experience, and assigns scores within each criterion (45). Test systems can be assigned as “waived complexity” under certain conditions, and these tests require no formal operator training or competency (45). In addition to CLIA-certified laboratories, the POC SARS-CoV-2 & Influenza A/B test is authorized for use in patient care settings that are CLIA waived (34). In conjunction with the fast turnaround offered by the Cobas Liat system and demonstrated in studies of the POC SARS-CoV-2 & Influenza A/B test in real-world settings (40, 46) or studies evaluating workflow or processing time (41, 47), our study confirms that the POC SARS-CoV-2 & Influenza A/B test is easy to use in CLIA-waived settings and offers results in a timeframe conducive for rapid patient management.

The strength of our multicenter study lies in the comprehensive nature of the variables assessed, encompassing a wide variety of patient characteristics likely to be encountered in healthcare settings. The study included a prospectively enrolled cohort of patients seeking care, which is representative of the intended-use population of the POC SARS-CoV-2 & Influenza A/B test.

Our study has some limitations. First, we did not ascertain the SARS-CoV-2 variants present in the samples. While the emergence of SARS-CoV-2 variants has the ability to affect the diagnostic performance of the test, previous studies have indicated that the POC SARS-CoV-2 & Influenza A/B test detects both wild-type and variants of concern, such as Alpha (B.1.1.7) or Omicron (B.1.1.529) (39, 47). Bioinformatic analysis to assess inclusivity showed that the POC SARS-CoV-2 & Influenza A/B test is predicted to bind all sequences available in the NCBI and GISAID databases as of July 2024. The dual target assay design helps to ensure that the assay is robust and safeguards against the emergence of variants that have the potential to affect assay performance and evade detection.

Second, the majority of positive influenza specimens, including all influenza B specimens, were obtained retrospectively, meaning that sample degradation was a possibility, although retrospective samples were only included if they had experienced only two freeze-thaw cycles. With some retrospective samples dating back to 2013, it is also possible that currently circulating variants were not well-represented within the sample. Analyses based on the age of the samples were not performed; therefore, it is unclear whether this was a factor in any observed discordances.

This analysis did not evaluate the cost of testing in comparison to centralized laboratory testing, although if assessed, the advantage of the speed of result availability should be given appropriate weight.

We recognize that comparing to a broader range of assays would be valuable in providing a comprehensive evaluation of performance relative to other commercial tests. However, our study design was in alignment with other studies in using the 6800/8800 SARS-CoV-2 assay, one of the most widely used assays in the US (38). We also incorporated three comparator tests to help mitigate potential bias.

Although beyond the scope of our analysis, we acknowledge that further assessments to understand the possible impact of the assay in terms of implementing infection control measures earlier and reducing the spread of disease via earlier detection would be of clinical interest.

### Conclusion

A POC RT-PCR test that combined measurement of SARS-CoV-2 and influenza subtypes with performance equivalent to routine centralized testing would provide a critical tool to improve the diagnosis and management of COVID-19 and influenza. We found that the performance of the POC SARS-CoV-2 & Influenza A/B test was comparable to centralized testing methods. Centralized testing has long been considered the gold standard. With the ease of use and equivalent performance of the POC SARS-CoV-2 & Influenza A/B test, we are moving toward a reality in which these assays are an equally valuable option. A study of the POC Cobas Influenza A/B test (an RT-PCR test that detects only the influenza analyte) in the emergency department found that POC testing for influenza was useful in improving several metrics, including the indication for treatment with neuraminidase in positive cases (48). It is relevant to note that SARS-CoV-2 and influenza coinfection can increase the risk of severe outcomes compared with those infected with SARS-CoV-2 alone, particularly for those coinfected with influenza A (49). Our study highlights the benefits of molecular multiplex POCTs to help improve the timely differentiation of respiratory diseases that share similar symptoms and support efforts to improve patient management.

### Assay disclaimers

The Cobas SARS-CoV-2 & Influenza A/B qualitative assay for use on the Cobas Liat system (here referred to as the POC SARS-CoV-2 & Influenza A/B test) was originally approved under Emergency Use Authorization (EUA) EUA201779 and has since been cleared and CLIA waived under K223591/CW220014, respectively, in the US and is CE-IVD marked in the European Union. Sample collection in the patient’s home is not approved in the US or European Union.

The Cobas SARS-CoV-2 & Influenza A/B qualitative assay for use on the Cobas 6800/8800 systems (here referred to as the 6800/8800 SARS-CoV-2 & Influenza A/B test) is authorized only for use under EUA in the US and is CE-IVD marked in the European Union. The assay is not approved for use in asymptomatic patients in the US or European Union. The assay is not approved for use as a POCT/near-patient test (NPT) in the US/ European Union. EUA approval for Cobas SARS-CoV-2 & Influenza A/B v2 MN 10033401190 was granted in November 2023, and the CE-IVD mark was granted in September 2023. Sample collection in the patient’s home is not approved in the US or European Union.

The Cobas SARS-CoV-2 qualitative assay for use on the Cobas 6800/8800 systems (here referred to as the 6800/8800 SARS-CoV-2 test) is authorized only for use under EUA in the US and is CE-IVD marked in the European Union. The assay is not approved for use as a POCT/NPT in the US/European Union. US FDA clearance (K240867) for 5800/6800/8800 Cobas SARS-CoV-2 Qualitative test was issued in February 2025; however the cleared test is not intended for self-collected nasal specimens in a healthcare setting. Sample collection in the patient’s home is not approved in the US or European Union.

Hologic Aptima SARS-CoV-2 assay was granted FDA EUA status in May 2020 (30), followed by an FDA 510(k) clearance in February 2025.

## Data Availability

The data sets generated during and/or analyzed during the current study are not publicly available due to patient confidentiality. Any access requests from qualified researchers should be submitted directly to the Ethical Committee of each participating study site.

## SUPPLEMENTAL MATERIAL

Supplemental material jcm.01459-24-s0001.docx Supplemental methods and Tables S1 to S9.

ASM does not own the copyrights to Supplemental Material that may be linked to, or accessed through, an article. The authors have granted ASM a non-exclusive, world-wide license to publish the Supplemental Material files. Please contact the corresponding author directly for reuse.
